# Polymer and Composite Membranes for Proton-Conducting, High-Temperature Fuel Cells: A Critical Review

**DOI:** 10.3390/ma10070687

**Published:** 2017-06-22

**Authors:** Eliana Quartarone, Simone Angioni, Piercarlo Mustarelli

**Affiliations:** Department of Chemistry of University of Pavia and INSTM, Via Taramelli 12, 27100 Pavia, Italy; eliana.quartarone@unipv.it (E.Q.); simone.angioni@unipv.it (S.A.)

**Keywords:** fuel cells, high temperature, membrane, polymer, PBI

## Abstract

Polymer fuel cells operating above 100 °C (High Temperature Polymer Electrolyte Membrane Fuel Cells, HT-PEMFCs) have gained large interest for their application to automobiles. The HT-PEMFC devices are typically made of membranes with poly(benzimidazoles), although other polymers, such as sulphonated poly(ether ether ketones) and pyridine-based materials have been reported. In this critical review, we address the state-of-the-art of membrane fabrication and their properties. A large number of papers of uneven quality has appeared in the literature during the last few years, so this review is limited to works that are judged as significant. Emphasis is put on proton transport and the physico-chemical mechanisms of proton conductivity.

## 1. Introduction

Sir W.R. Grove reported the concept of fuel cells (FC) more than 150 years ago. Since then, many kinds of FCs have been reported in the literature and developed at the industrial level [1]. Among them, Polymer Electrolyte Membrane (or Proton Exchange Membrane) fuel cells, abbreviated as PEMFCs, are the most common ones. If fully successful, they could solve most of the energy issues of the future. PEMFC variants include low temperature PEM fuel cells (LT-PEMFCs), high temperature PEM fuel cells (HT-PEMFCs) and direct alcohol fuel cells (DAFC). In particular, HT-PEMFCs operating in the temperature range from 120–200 °C [2,3,4,5] are of wide interest for their large-scale application in automobiles, as well as in combined heat and power systems (CHP) and auxiliary power units (APU). The main advantages of HT-PEMFCs with respect to LT-PEMFCs (working at about 80 °C with perfluorosulphonic membranes as the electrolyte) are: (i) improved tolerance to catalyst poisoning [6,7]; (ii) enhanced electrode kinetics [8]; (iii) easier heat dissipation and water management; (iv) better thermodynamic quality of the produced heat.

Together with these advantages, there are also several challenges associated with operation at higher temperature. Among these, the most important are: (i) increased degradation rates due to higher operating temperatures; and (ii) increased start-up time.

HT-PEMFCs generally use polybenzimidazole (PBI)-based membranes and other aromatic amines activated with phosphoric acid (PA) as the electrolyte. Other membranes based on aromatic/polyether systems, as well as blended/composite moieties have been reported in the literature. In this critical review, we will discuss the most important advances in the field, by also giving emphasis to some recent advances in understanding the proton transport mechanisms of PA-based systems.

## 2. PBI-Based Membranes

Polybenzimidazole is indeed the most important polymer used for the fabrication of membranes for HT-PEMFCs [9]. After the pioneering works of Savinell and Wainright at Case Western University [10,11,12], PBI membranes were developed by several groups [13,14,15,16]. Preparation methods include polymerization in polyphosphoric acid (PPA) [17], casting from methane sulphonic acid [18,19], and microwave assisted organic synthesis [20,21].

**Scheme 1:**
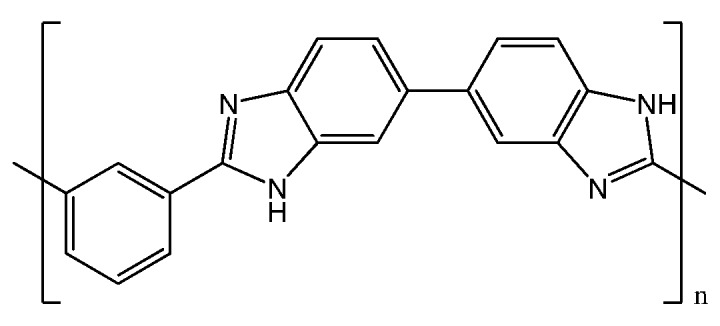
Chemical structure of poly[(2,2′-(m-phenylene)-5,5′-(bibenzimidazole)].

As stated, PBI-based membranes show high proton conductivity if properly doped with strong acids such as H_3_PO_4_. In fact, PBI is a basic polymer, which dissociates PA releasing protons, as sketched by the following reaction:

H_3_PO_4_ + PBI → H_2_PO_4_^−^ + PBI-H^+^

where the equilibrium constant is about K = 1.17 × 10^3^. H^+^ ions can migrate through the polymer backbone by means of hydrogen bonds, in this case assisted by phosphate anions. The exact transport mechanisms are quite complex, and also depend on the amount of PA absorbed into the polymer matrix. This point will be carefully addressed in the following sections. The most investigated PBI membrane used as an electrolyte for HT-PEMFCs is indeed poly[(2,2′-(m-phenylene)-5,5′-(bibenzimidazole)], whose monomer structure is reported in Scheme 1 and labelled as PBI_4N, which includes two basic nitrogen units. It is obtained by reacting 3,3′-diaminobenzidine and dicarboxylic acid.

Molecules containing benzimidazole moieties are generally characterized by high reactivity, which allows for a rich chemistry. Therefore, by changing the chemical nature of the starting monomers it is possible to modify the polymer backbone in order to modulate the physico-chemical properties of the final membrane, including thermo-oxidative stability, mechanical performances, methanol permeability, polymer solubility, and film processing. In this frame, polymers including functional groups based on ether, ketone, sulphone, and fluoroalkyl units appeared in the literature [7,18,19]. PBIs can be also cross-linked to improve the mechanical strength of the doped membranes, which is a critical issue for practical operation. Several types of crosslinking processes have been tested, including covalent, ionic, and mixed ionic-covalent crosslinking [22,23]. However, if the membrane toughness increases, the polymer solubility in the conventional casting solvents decreases as well.

Table 1 reports some PBI-based polymers obtained by changing one of the monomers. These substitutions allow modifying the physico-chemical properties (e.g., basicity), simply by playing with the number of the nitrogen atoms in the monomer and their distribution along the polymer backbone [24]. By this way it is also possible to reduce acid leaching, therefore enhancing the permanent conductivity of the membrane [25].

**Scheme 2:**
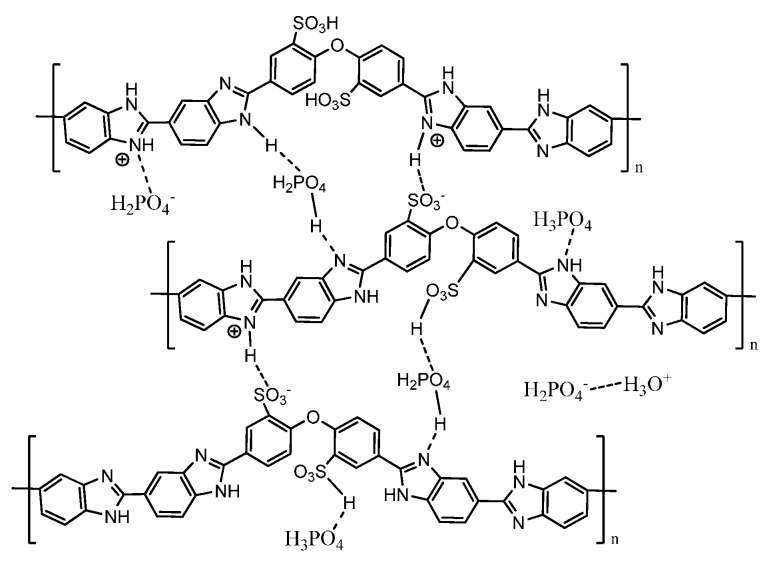
Non-covalent interactions in sulphonated PBI membranes (taken from Ref. [28]).

Another important modification of the PBI backbone is sulphonation. In particular, aryloxy-based PBIs were recently investigated because they may be sulphonated with mild synthetic routes, due to the electron-donor properties of the ether bridges [26,27]. The presence of protogenic groups such as –SO_3_H combines the benefits of the low- and high-temperature membranes, confers low swelling properties and mechanical strength, and remarkably improves the polymer solubility in safer solvents. Here, one critical issue is the preparation of polymers with a reproducible sulphonation level, and ordered distribution of the protogenic units. A good approach to solve the problem consists of the polymerization of sulphonated monomers, based on benzimidazole units including aryloxy-spacers, which are particularly electron-rich groups and are consequently more easily sulphonable [20]. On the other hand, sulphonation may increase the non-covalent interactions among the polymer chains and the PA molecules (see Scheme 2), thereby increasing the stiffness of the membrane and reducing the proton mobility [28].

**Scheme 3:**
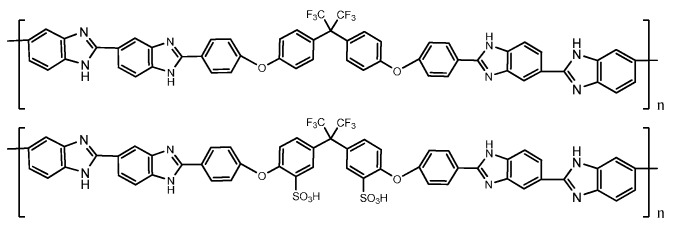
Structure of F_6_-oxyPBI (**upper**) and F_6_-oxyPBI-2SO_3_H (**lower**) (taken from Ref. [21]).

A possible mitigation of this problem is given by the sulphonation of fluoro-oxy PBI membranes (Scheme 3). The presence of fluorine atoms, in fact, confers better organo-solubility to the system due to the flexibility of the –C(CF_3_)_2_– group, higher chemical stability against oxidation, and also improved capability as a methanol barrier [29,30,31]. Sulphonated fluoro-oxy PBI membranes can be easily obtained by microwave-assisted synthesis [21].

**Figure 1:**
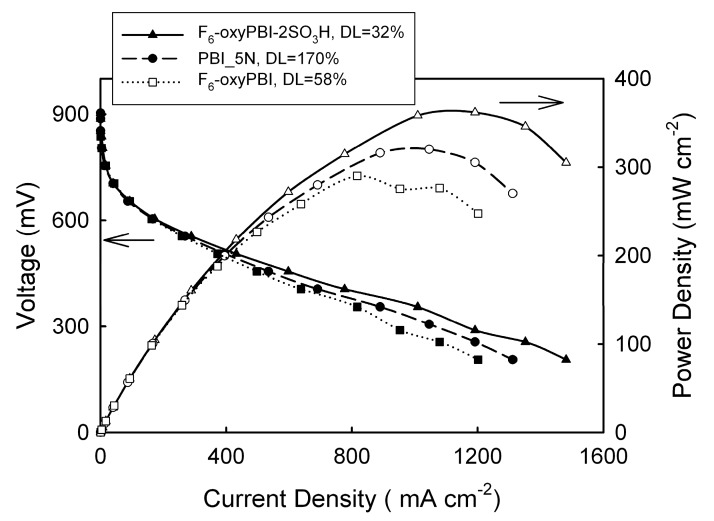
Polarization curves of MEAs based on fluoro- and sulfonated fluoro-oxyPBI membranes at 150 °C and without any external humidification. The data of PBI_5N are shown for comparison. DL: doping level (taken from Ref. [21]). DL is given by the relationship.

In spite of doping levels and conductivity values lower than those of standard PBI membranes, very promising fuel cell performances were obtained for the membrane-electrode-assemblies (MEAs) based on the F_6_-oxyPBI-2SO_3_H membrane. In particular, power densities in excess of 360 mW cm^−2^ were obtained at 150 °C without any external gas humidification in a H_2_/air configuration (see Figure 1). MEA based on the fluoro-sulphonated PBI did not undergo degradation during 800–1000 h.

$$DL_{PA}=\frac{W_{PA}}{W_{dry-PBI}}\%$$

where *W_dry-PBI_* is the weight of the membrane before the acid uptake and *W_PA_* is the mass of the absorbed H_3_PO_4_.

PBI-based membranes doped with H_3_PO_4_ have several drawbacks. For example, it was reported that they could bleed out a part of the phosphoric acid molecules when the operation temperature of the fuel cells is below 100 °C, and the water produced by the fuel cell operation can react with H_3_PO_4_ [32]. Moreover, the released H_3_PO_4_ can damage the fuel cell ancillary systems through corrosion processes. Another significant problem of pure H_3_PO_4_-doped PBI membranes is given by the chemical degradation of the polymer [33]. Several strategies were proposed to overcome these problems, including: (i) application of acid-base concepts; (ii) fabrication of composites and nanocomposites; (iii) covalent cross-linking. These points will be discussed in the following sections.

## 3. PBI-Based Acid-Base Blends

**Figure 2:**
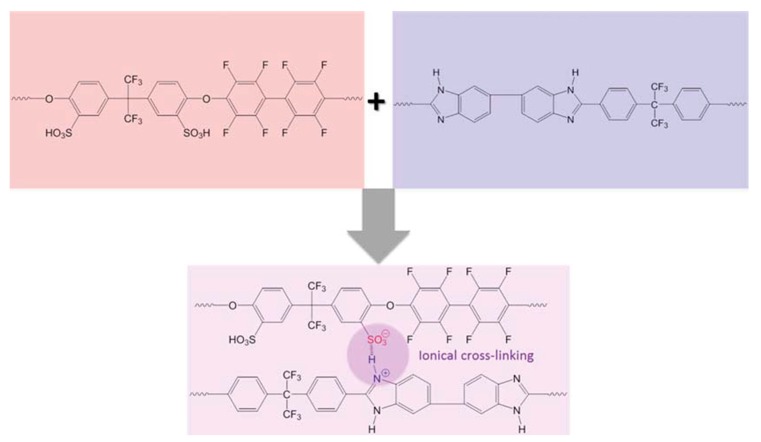
Acid-base polymer cross-linking (from Ref. [34]).

This concept is based on the blending of a basic polymer with an acidic one, where acid–base, non-covalent cross-links are formed by proton transfer from the acidic group to the basic one (see Figure 2). This approach was proposed early on by Kerres et al. for the fabrication of membranes working at low temperature [34]. Here, the basic polymer was used as a cross-linker for the cation-conducting membranes. By cross-linking the cation exchange polymer, it was possible to reduce the water uptake of the membrane, which led to the improvement of its mechanical stability [35].

It was reported [36] that ionic cross-linking of the membranes also led to an improvement of thermal stability and resistance to radical attack (measured by reduced weight loss after immersion in H_2_O_2_ solutions, compared to the pure acidic polymers). The ionic conductivity of blend systems can be varied by variation of the molar ratio between the acid and the basic moieties.

The acid–base blend concept was firstly applied to HT-PEMFCs by Hasiotis et al. [37], who combined polybenzimidazole with sulphonated polysulphones (SPSF), followed by doping with phosphoric acid. In these blends the acidic polymer works as the macromolecular cross-linker, whereas the phosphoric acid provided proton moieties for the ionic transport. The conductivity of SPSF was of the order of 10^−3^ S cm^−1^. In the case of blends of PBI and SPSF, it was found to be higher than 10^−2^ S cm^−1^.

**Figure 3:**
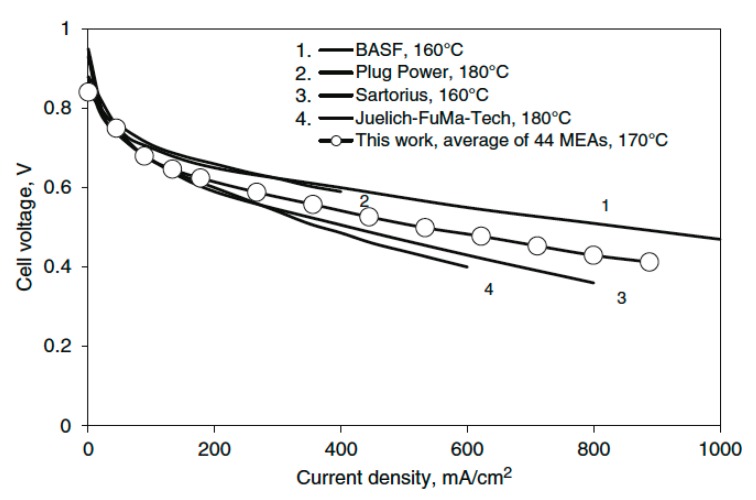
Fuel cell performances of commercial membranes compared with a blend one (taken from Ref. [34]).

After that, the Kerres group tested a large number of acid-base blend couples [34]. Figure 3 shows the comparison of the fuel cell performances of some commercial membranes with a blend composed of PBI Celazol^®^ (poly(2,20-m-phenylene-5,50-bibenzimidazole, m-PBI, named as B1, PBI Performing Products Inc., Charlotte, NC, USA) and polysulphone Udel^®^ (Solvay, Milan, Italy) sulphonated in the bisphenol A section (named S1). The B1/S1 ratio was 70:30 wt %.

Recently, blend membranes obtained with fluorinated and aryloxy-PBI as the basic component were also reported [38,39]. In contrast to pure PBI ones, these membranes exhibited long-term stability in PA at 130 °C. Ionic cross-linking between the acid and base blend polymers improved the stability and integrity of the membranes. MEAs based on these blend membranes showed good fuel cell performance at practically relevant operation conditions.

A new method to crosslink PBI, starting from ionically cross-linked acid/base blend membranes, was also reported [40]. By heating the membrane to temperatures above 200 °C, a Friedel-Crafts reaction between sulfonic acid groups and electron-rich phenyl groups took place, which covalently cross-linked the acid and base components in the blend by chemically stable aromatic sulphone bonds. The effects of curing temperature and time on some membrane properties, including solubility, phosphoric acid uptake, and mechanical strength were reported. The membrane was tested in a fuel cell showing a good performance.

## 4. PBI Composite Membranes

The addition of a second (and even a third) component to PBI, to give a composite or a nanocomposite membrane, may lead to improvements concerning water (or PA) uptake and retention, proton conductivity, thermal and mechanical properties, fuel cell performances, and durability. Several categories of materials can be added as the filler phase, including oxides, heteropolyacids and their salts, pyrophosphates, carbon-based materials, reinforcing polymer phases, and ionic liquids. Here we will limit our discussion to inorganic phases and polymers.

### 4.1. Hygroscopic and Non-Hygroscopic Oxides

The most important oxide to be considered is indeed silica, SiO_2_, which in most cases is modified (organically or inorganically) in order to improve the properties of the membrane. The incorporation of (nano)silica is often performed by in situ sol-gel reactions during membrane casting [41].

**Figure 4:**
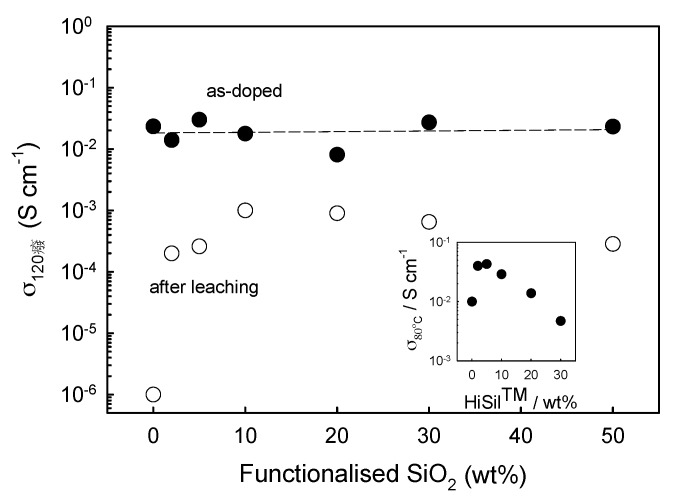
Proton conductivity vs. the filler content at 120 °C and 50% relative humidity, both for the as-prepared membranes and after leaching. The inset shows the proton conductivity behaviour when a commercial nanometer-scale silica is used as the filler (taken from Ref. [42]).

We reported [42] the fabrication of silica functionalized with imidazole by means of a standard sol-gel process, starting from tetraethoxysilane (TEOS) and N-(3-triethoxysilylpropyl)-4,5-dihydroimidazole. The functionalized silica (Im-SiO_2_) was added to a PBI solution and then cast to produce the membrane. Figure 4 shows the behaviour of the proton conductivity of the membrane as-prepared and after forced leaching of PA. Our results show that 10 wt % of filler gave ~10^3^ increase of the permanent conductivity. Issues related to the hygroscopic nature of silica, as well as to its long-term resistance in contact with PA, must be taken into account.

A similar procedure was used to prepared PBI–montmorillonite nanocomposite membranes, from an organosoluble, fluorine-containing copolymer [43]. Later, we compared the role of Im-SiO_2_ with that of some mesostructured silicas, namely SBA-15 [44,45] and MCM-41 [46].

The group of Lobato fabricated TiO_2_-based composite PBI membranes, showing that there was a significant improvement of the fuel cell performances [47,48,49]. The addition of modified TiO_2_ and SiO_2_ nanoparticles to a poly[2,2-5,5-(m-pyrazolidene)-bibenzimidazole] (PPBI) was also reported [50]. The modification of both SiO_2_ and TiO_2_ nanoparticles was performed by radical polymerization of vinyl monomers such as sulfonated polyvinylbenzene (PSV) or poly-vinylimidazole (PVI) on the surface of the nanoparticles.

### 4.2. Heteropolyacids, Salts, and Phosphates

The key issue at the basis of these inorganic materials is the attempt to increase the conductivity of the membrane at lower acid contents, while keeping or even improving the mechanical properties and chemical stability.

Heteropolyacids (HPAs) display high conductivity and strong acidity and are generally surrounded by a large number of water molecules, making them suitable for operation under anhydrous conditions. The HPAs most frequently used as fillers in proton-conducting systems are: phosphotungstic acid (H_3_PW_12_O_40_, PWA) [51], phosphomolybdic acid (H_3_PMo_12_O_40_, PMA) [52], and silicotungstic acid (H_3_SiW_12_O_40_, SWA) [53]. Whereas high conductivity levels can be reached only with PA addition, HPAs’ high water affinity improves their CO tolerance [54].

HPAs can be modified by partial substitution with cesium (CsHPA) in order to improve their surface acidity [55]. This substitution reduces the water solubility of the salt and increases its surface area, thereby maximizing the interaction with the polymer matrix. This also increases the membrane conductivity, whereas the silica-based salt reinforces the mechanical properties of the membrane.

Zirconium (Zr(HPO_4_)_2_.nH_2_O, ZrP) [14] and boron (BPO_4_) [56] phosphates were also used in order to fabricate PBI composite membranes. These materials, chiefly ZrP, lead to enhanced proton conductivity and improved resistance to methanol permeation.

Tin-based pyrophosphates were also used as fillers to prepare composite membranes. These materials show high conductivity above 150 °C under anhydrous conditions. Promising results were reported concerning the fuel cell performance [57,58].

### 4.3. Carbon-Based Fillers

Among the carbon-based materials, the most important are functionalized multi-walled carbon nanotubes (MW-CNTs) and graphene oxide. The fabrication of PBI composites with CNTs must be carefully evaluated because of their electronic conductivity, which may cause short circuits in the cell. However, π–π interactions between the PBI strands and the side-walls of CNTs, as well as proper nanotube functionalization, can make this problem manageable. Although CNT-PBI composite membranes showed improvements of the mechanical strength, the proton conductivity may be reduced [59].

Phosphonated MWCNTs were added to a PBI membrane (pCNT-PBI) up to 2.5 wt %, and the composites were then doped with PA [60]. A similar doping level was obtained for both pristine and composite-based PBI membranes. The pCNT-PBI membranes showed higher proton conductivity than the pristine PBI and non-phosphonated CNT-PBI membrane. These results were rationalized in terms of a network formed by the phosphonic groups in the CNT, which facilitated proton conduction.

**Figure 5:**
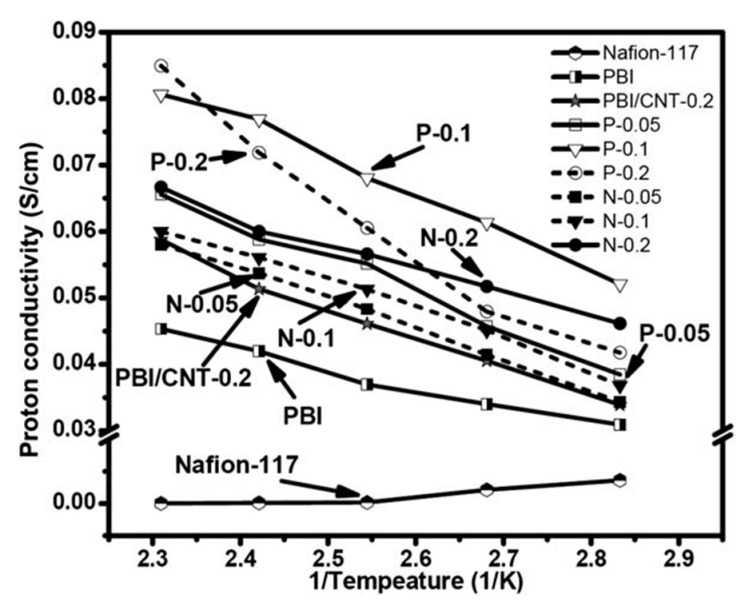
The proton conductivity of Nafion-117, PA-doped PBI, PBI/MWCNT–PBI, and PBI/MWCNT–Nafion composite membranes at 80–160 °C without humidification. The data measured with the composite membrane made of non-functionalized MWCNT (0.2 wt %) and PBI are also shown for comparison (taken from Ref. [61]).

Suryani et al. [61] prepared Nafion™- and PBI-functionalized MWCNT and used them as fillers in the preparation of MWCNT-PBI composite membranes. The proton conductivities of the PBI-MWCNT Nafion™ membranes were higher than that of the PBI membrane, but lower than the values of the PBI:MWCNT-PBI composite ones (see Figure 5). However, after phosphoric acid doping, the sulphonated PBI membranes showed a higher proton conductivity compared to the non-sulphonated PBI. The fuel cell tests revealed that the modification of PBI with 0.2 wt % of MWCNT-PBI enhanced single cell performance by 32%.

Graphite oxide (GO) was also used as a filler to prepare PBI composite membranes [62]. Whereas GO by itself is an electronic insulator, the presence of acidic functional groups, such as carboxylic acid, can provide pathways for the hopping of protons. Xu et al. [63] prepared GO-PBI and sulphonated graphite oxide (SGO-PBI) (2 wt % of filler) composite membranes. The in-plane conductivity of the composite membrane was higher than that of the pristine PBI membranes, measured under the same operational conditions, with very low PA doping levels. The fuel cell tests at 175 °C gave a peak power density of 0.14, 0.31, and 0.43 Wcm^−2^ for the PBI, GO-PBI, and SGO-PBI membranes, respectively.

### 4.4. Polymer Phases

The concept of polymer phase reinforcement to improve mechanical strength and reduce gas crossover was proposed early on for membranes based on perfluorinated polymers, e.g., Nafion™ [64]. Later the concept was applied to fuel cell technology by using polytetrafluoroethylene (PTFE) as the reinforcing phase [65].

**Figure 6:**
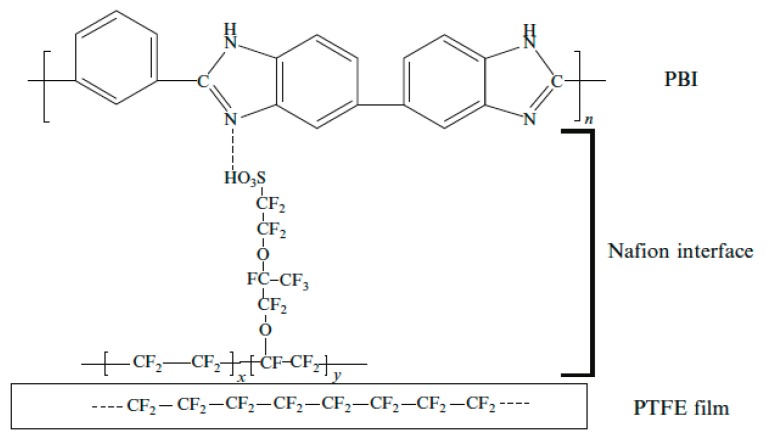
The role of Nafion™ as the coupling agent in PTFE-PBI composite membranes (taken from Ref. [67]).

Similar to the preparation of Nafion™/PTFE systems, PBI/PTFE composite membranes could be prepared by the impregnation of the porous PTFE support film with a PBI solution, followed by solvent evaporation. Because of the low chemical compatibility of PTFE and PBI, it is mandatory to perform proper pre-treatments in order to activate PTFE, thereby obtaining good interfacial properties between PTFE and PBI. Two different methods were reported in the literature: chemical activation [66] and coupling agent pre-treatment [67]. In chemical activation, the surface of the PTFE films must be activated by immersion in H_2_SO_4_/H_2_O_2_ solutions, followed by immersion in basic NaOH, and then in DMAc. Within the latter method, Nafion™ was used as the coupling agent. The porous PTFE thin film was immersed in a 0.7 wt % Nafion™/DMAc solution to form a thin film of Nafion™ on the surface of a PTFE fiber. Figure 6 shows the role of Nafion™ as the interface coupling agent for PBI and PTFE. PTFE-reinforced membranes showed improved fuel cell performances with respect to PBI ones.

### 4.5. PBI Application in Stacks

It is well known that beside the loss of phosphoric acid, the major problems affecting the lifetime of the PBI-based fuel cells likely are catalyst poisoning, polymer oxidative degradation, and catalyst agglomeration/sintering or dissolution [9]. Recently, the Lobato group reported the feasibility of a 150 cm^2^ HT-PEMFC stack [68]. The fuel cell stack was prepared using three 50 cm^2^ PBI-based MEAs, and the authors compared pure PBI and modified membranes contained 2 wt % micro-sized TiO_2_ as a filler. Long-term tests were performed in both constant and dynamic loading modes. The fuel cell stack with 2 wt % TiO_2_ composite PBI membranes exhibited an irreversible voltage loss of less than 2% after 1100 h of operation. In addition, the acid loss was reduced from 2% for the fuel cell stack with pure PBI to 0.6% for the fuel cell stack with modified membranes. Moreover, the fuel cell stack also exhibited very rapid and stable power and voltage output responses under dynamic load regimes.

## 5. Other Polymer Membranes

**Figure 7:**
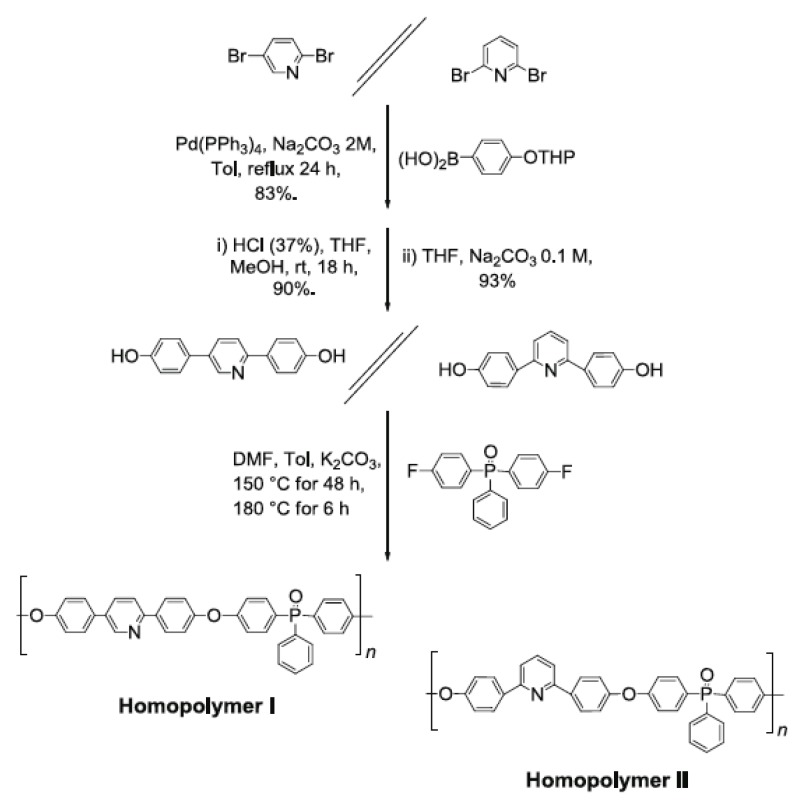
Rigid aromatic polymer based on pyridine. Reaction schemes proposed in Ref. [68].

Whereas PBI is indeed the most important player in HT-PEMFCs technology, other polymers and their combinations (also in the case with PBI) were proposed during the last decade. In 2003 the Kallitsis group proposed the use of pyridine units as basic moieties in a rigid aromatic polymer [69]. Here, phosphinoxide moieties were also included in the polymer structure in order to improve the solubility and to create more interaction sites with the phosphoric acid doping agent (see Figure 7).

In the recent years, many polymers and co-polymers were synthesized by the same group, including chemical moieties such as SO_2_, CH_3_, SO_3_Na, and OH [70]. Some of these materials demonstrated significant advantages compared to pure PBI membranes, concerning their manufacturing properties, operating conditions, and fuel cell performance. In particular, they showed good film-forming properties, long lasting mechanical and chemical stability, good proton conductivity (up to 10^−1^ S cm^−1^), and operating temperatures between 170–210 °C.

**Figure 8:**
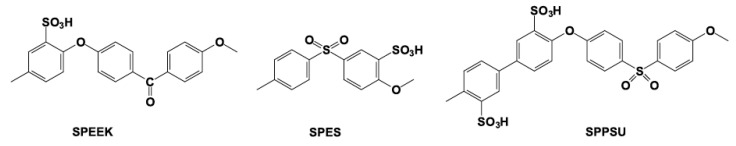
Chemical formulas of some sulphonated aromatic polymers.

Sulphonated poly(ether ether ketone) (SPEEK), together with other sulphonated aromatic polymers such as polyphenylsulfone (PPSU) and polyethersulfone (PES) (see Figure 8), were widely employed during the last decade to fabricate membranes operating in the range from 90–120 °C [71].

SPEEK can be obtained by post sulphonation of commercial PEEK, or by polymerization of monomers bearing sulphonic acid groups [72]. Whereas PEEK is a semi-crystalline polymer, chemically and thermally stable due to the presence of aromatic rings, SPEEK is generally amorphous. Its functional properties strongly depend on the history of ionomeric membranes, including solvent, treatment and measurement conditions, and equilibration times. The degree of sulphonation (DS) is defined as the number of sulfonic acid groups per repeat unit. Membranes with DS > 0.3 are soluble in DMSO, DMAc, and NMP. If DS > 0.7 the polymers are also soluble in warm water. For SPEEK membranes cast from DMAc or NMP with DS = 0.42 and 0.49, hydration values, λ, defined as the number of water molecules per sulphonic acid group, around 45 and 70 at 100 °C were obtained, respectively [73]. Proton conductivity values around 2.5 10^−2^ S cm^−1^ at 100 °C and 85% RH for SPEEK with DS **=** 0.89 were reported [74].

SPEEK-based composite membranes with inorganic [75], or organic-inorganic moieties (Organically-Modified Silicates, ORMOSIL) [76], were proposed by the Licoccia group. These materials showed promising properties for use in fuel cells.

Finally, some significant results must be put into evidence concerning non-polymeric membranes. In^3+^-doped SnP_2_O_7_ membranes were reported more than 10 years ago by Nagao et al. in the temperature range from 100 to 300 °C [77]. The proton conductivity was higher than 10^−1^ S cm^−1^ between 125 and 300 °C. The resulting fuel cell exhibited a reasonable power density of 264 mW cm^−2^ at 250 °C, together with a tolerance toward 10% CO and good thermal stability under non-humidified conditions. More recently, Lee et al. [78] demonstrated that fuel cells based on quaternary ammonium (QA)-biphosphate ion-pair-coordinated polyphenylene (PA-doped QAPOH) PEMs could avoid the limitations of Nafion™ and PBI-based fuel cells, enabling operation under a wide range of conditions. These PEMs can conduct protons through stable ionic pair complexes and enable fuel cell operation at temperatures from 80 to 180 °C.

## 6. Proton Conductivity and Transport Mechanisms

The proton conductivity of acid-doped PBI membranes is strongly dependent on the physico-chemical parameters such as temperature and relative humidity, as well as on the acid doping level, which, in turn, depends on the molar concentration of the doping solution [14,79]. Let us start from the peculiar nature of the proton ion. Due to its very small dimensions (~10^−15^ m), the proton can approach neighbouring atoms or ions forming strongly bonded entities. In particular, in aqueous media, the proton can form multinuclear charged species such as H_3_O^+^, NH_4_^+^, H_2_PO_4_^+^, etc. Moreover, it can form extended hydrogen-bonding (HB) networks. The unique properties of protons translate into two possible mechanisms for ion conduction: (i) vehicular, i.e., motion assisted by carrying molecule(s); and (ii) Grotthuss, i.e., jumping from one site to another one along a HB chain.

Concerning the conductivity of PBI-based membranes, several mechanisms for proton transport were proposed [9]:

(1) direct hopping along the nitrogen sites of PBI chains, which is relevant only for non-doped PBI;
(2) hopping from the N-H sites to phosphoric acid anions. This contribution is relevant for *n* = [H_3_PO_4_]/[BI] < 2;
(3) hopping along the phosphoric acid anions (*n* > 2). This term is associated to free acid, and can contribute a strong increase of the conductivity;
(4) hopping via water molecules, concurrent with the previous one, which is significant at high temperature.

Whereas, in most cases the Arrhenian behaviour of the conductivity calls for prevalent proton hopping [24], we recently showed that acid-doped PBI_5N membranes exhibit a Vogel–Tammann–Fulcher (VTF) conduction behaviour, where proton hopping is coupled with the segmental motion of the polymer chains [80].

In order to obtain a deep understanding of the proton transport mechanisms in these systems, it is mandatory to address the situation in pure H_3_PO_4_ and then in mixed H_3_PO_4_-H_2_O solutions as model systems. Both these tasks were investigated by the Kreuer group. Proton transport in pure H_3_PO_4_ was investigated by ab initio molecular dynamics [81]. They showed that fast proton transport in hydrogen-bonded systems involves a structural diffusion mechanism, where intramolecular proton transfer is driven by specific HB rearrangements in the surrounding environment. Whereas, in aqueous media, the excess charge defects move through local hydrogen bond rearrangements, the strong and polarizable HBs in phosphoric acid will produce coupled proton motion and a significant dielectric response of the medium, thereby leading to the formation of extended, polarized hydrogen-bonded chains (structural diffusion). The interplay between these chains and a frustrated HB network is at the basis of PA high proton conductivity. Here, the concept of “frustration” is related to the imbalance between proton donors and acceptors. In particular, “frustrated protons” are protons covalently bonded to HB-accepting oxygens that cannot themselves participate to HBs because of a scarcity of acceptors. Frustrated protons readily form new HBs, thereby stabilizing excess charge, which produce irreversible charge separation and thus conduction.

**Figure 9:**
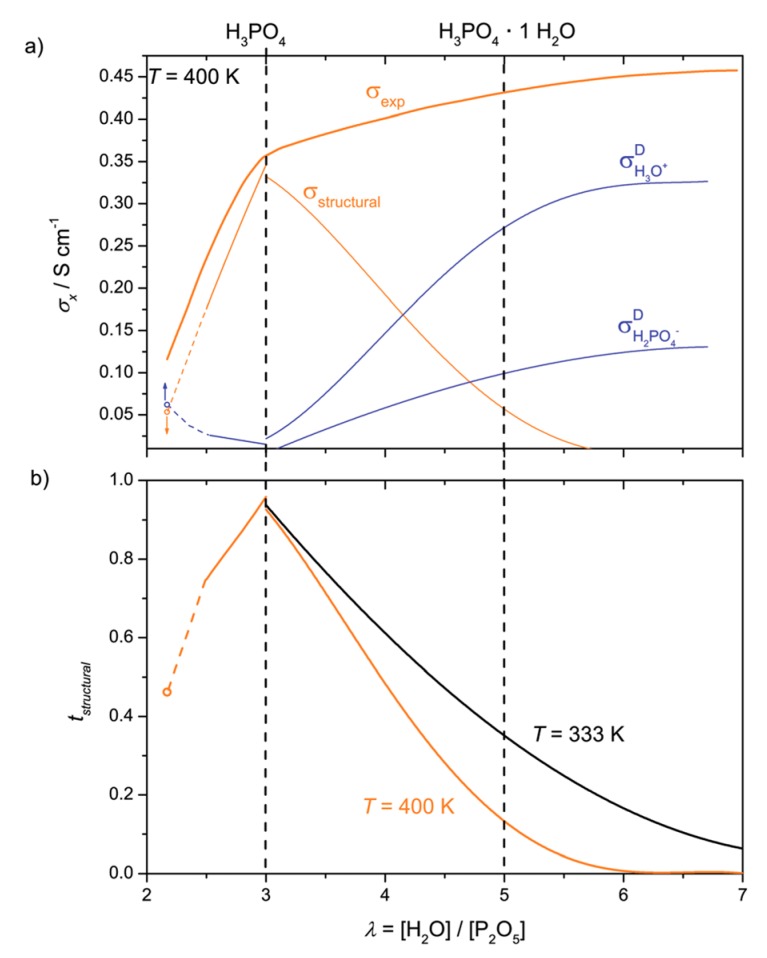
(**a**) Total proton conductivity, *σ*_exp_, of phosphoric acid as a function of *λ* and its contributions from proton structural diffusion, *σ*_structural_, and ionic transport associated with the hydrodynamic background *σ*_vehicle_; (**b**) The corresponding structural transference numbers at different temperatures (taken from Ref. [82]).

A recent paper [82] discussed the P_2_O_5_-*λ*H_2_O system for *λ* ≤ 100 (here H_3_PO_4_ corresponds to *λ* = 3). The authors showed that structural and vehicular transport are predominant for *λ* < 3 and *λ* > 7, respectively. For *λ* in the range from 3–7, there is a transition region where the structural diffusion is progressively reduced because of frustration decrease. These results underlined the singularity of structural diffusion in PA and its sensitivity against any perturbation (see Figure 9).

The specific case of H_4_P_2_O_7_ was previously investigated by coupling *ab initio* molecular dynamics simulations and NMR spectroscopy [83]. Here, the central oxygen of the molecule was found to be excluded from the HB network, which has two consequences: (i) the acidity of H_4_P_2_O_7_ is higher compared to H_3_PO_4_; and (ii) the condensation reaction leads to a minor decrease in HB network frustration, which is thought to be one of the features enabling high proton conductivity. At T = 160 °C, the hopping mechanisms were estimated to give a conductivity contribution of about 0.1 S cm^−1^, which is about half of the observed ionic conductivity (*σ* ≈ 0.2 S cm^−1^).

Finally, the transport properties of PA/benzimidazole mixtures, intended as a model system for PBI-based membranes, were investigated [84]. NMR results demonstrated that benzimidazole is acting as a strong Brönsted base with respect to PA. As nitrogen atoms of benzimidazole are fully protonated, and there is a low rate of proton exchange with phosphate moieties, proton transport must take place within the HB network of PA. The subtraction of protons from PA leads to a strong reduction of the proton diffusion coefficient with respect to pure PA. This effect also reduces HB network frustration and, consequently, structural proton diffusion, PA acidity, and hygroscopicity. The reduced water uptake gets along with the low electroosmotic water drag, which is likely the reason why PBI–PA membranes display better fuel cell performances than other systems containing PA as the electrolyte, even if these show better conductivity properties.

## 7. Conclusions

In this paper, we discussed the most significant advances in the field of membranes for high temperature polymer fuel cells. Although alternative polymer matrices were proposed during the last few years, polybenzimidazole-based moieties and their blends and composites are still the best choice for the fabrication of efficient and durable membranes. The excellent properties of PBI in terms of proton conductivity are likely related to the unique physico-chemical properties of the H_3_PO_4_ doping agent, which were carefully addressed in the last few years.

In the future, it will be mandatory to perform careful multiscale (multiphysics) modelling of the membranes and corresponding MEAs, in order to improve water and thermal management, as well as to optimize the chemical and electrochemical stability for long-term operation.

## Figures and Tables

**Table 1:** Polymers names: ***PBI_4N*:** Poly-2,2′-(m-phenilene)-5,5′-bibenzimidazole. ***PBI_5N_2,5*:** Poly-2,2′-(2,5-pyridine)-5,5′-bibenzimidazole. ***PBI_5N_2,6*:** Poly-2,2′-(2,6-pyridine)-5,5′-bibenzimidazole. ***PBI_6N_bipy*:** Poly-2,2′-(2,2′-bipyridine-5,5′)-5,5′-bibenzimidazole). ***PBI_6N_pyra*:** Poly-2,2′-(3,5-pyrazole)-5,5′-bibenzimidazole. *η*: intrinsic viscosity. MW*_mon_*: monomer molecular weight. *DP*: degree of polymerization. *MW_pol_*: polymer molecular weight.

	Polymer	η (dL/g)	MW_mon_	DP	MW_pol_
PBI_4N	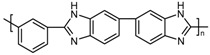	0.7	308	98	30,352
PBI_5N_2,6	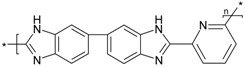	1.7	309	300	92,840
PBI_5N_2,5	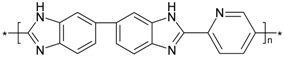	0.5	309	64	19,864
PBI_6N_bipy	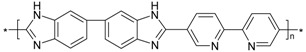	1.0	386	123	47,574
PBI_6N_pyra	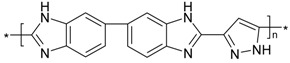	n.a.	298	n.a.	n.a.
